# EBV Infection Unmasking a Choledochal Cyst in an Infant

**DOI:** 10.1155/2019/7320109

**Published:** 2019-01-29

**Authors:** Hassan El Khatib, Batoul Kawtharany, Diyaa Mohammad, Mohammad Siblini, Nahida El-Rifai

**Affiliations:** ^1^Department of Pediatrics, Makassed General Hospital, Beirut, Lebanon; ^2^Department of Surgery, Makassed General Hospital, Beirut, Lebanon

## Abstract

Hepatic involvement is common in acute Epstein–Barr virus (EBV) infection in children. It usually manifests as a transitory elevation of transaminases in up to 80% to 90% of patients, and they normalize by 2 to 6 weeks. A cholestatic pattern with elevated gamma-glutamyl transferase (*γ*GT) and alkaline phosphatase (ALP) is common, in up to 60% in young adults. However, jaundice is very rare occurring in only 5% of pediatric patients. We report here an 8-month-old female with EBV infection who developed obstructive jaundice 2 weeks after the initial infection. Radiologic investigations were compatible with choledochal cyst type IVa complicated by stone formation in the common bile duct. In case of clinical exacerbation or nonamelioration of liver function tests in EVB infection, another diagnosis should be addressed. This highlights the importance of close follow-up in these patients in order not to miss a serious underlying condition such as choledochal malformation.

## 1. Introduction

Hepatic involvement is common in acute Epstein–Barr virus (EBV) infection in children. It usually manifests as a transitory elevation of transaminases in up to 80% to 90% of patients that normalizes by 2 to 6 weeks. A cholestatic pattern is common, in up to 60% in young adults. However, jaundice is very rare occurring in only 5% of patients [1]. A persistent cholestatic pattern should raise the suspicion of an underlying disorder such as choledochal cyst (CC).

We report here an 8-month-old female with EBV infection who developed obstructive jaundice 2 weeks after the initial infection. Radiologic investigations were compatible with choledochal cyst type IVa complicated by stone formation in the common bile duct.

## 2. Case Presentation

EK, an 8-month-old female, was born at 35 weeks by C-section due to preeclampsia with intrauterine growth restriction (Birth weight: 1.25 Kg). She stayed in the intensive care unit for 25 days for nutritional support and was discharged home in a good condition.

She was doing well until the age of 8 months when she presented to our Emergency Department with 1 day duration of high-grade fever; irritability; nonbilious, nonprojectile vomiting; multiple episodes of watery, nonbloody diarrhea; and PO intolerance. Upon physical examination, the patient was irritable and ill-looking with mottled skin and moderate dehydration. The anterior fontanel was soft nonbulging with bilateral erythematous tympanic membrane and pharynx. Her abdomen was soft, nondistended, and nontender, with no hepatomegaly. The rest of the physical examination was unremarkable. Lumbar puncture was done and was negative. The urine and CSF cultures were negative, and subsequently, the antibiotics were discontinued. Complete blood count showed hemoglobin: 12.2 g/dl, hematocrit: 38.2, WBC: 9300, bands: 10, neutrophils: 59, lymphocytes: 14, platelets: 364000, and C-reactive protein: 1.06 mg/dl. The liver function test was as follow: SGPT: 526 U/L (Nl < 40 IU/L), SGOT: 340 U/L (Nl < 40 IU/L), bilirubin (T/D): 2.56/2.24 mg/dl, alkaline phosphatase (ALP): 443 U/L (Nl: 150–420 U/L), gamma-glutamyl transferase (GGT): 662 U/L (Nl < 32 U/L), and albumin 4.38 g/dl (Nl: 3.5–5 g/dl). Serology for hepatitis A, B, and C and HIV was negative. Stools analysis was negative for rotavirus and adenovirus. Stools culture did not grow any pathologic bacteria. The anti-EBV viral capsid antibodies IgM was positive (IgM > 1.64 (Nl < 1.1)). Ultrasound of abdomen was normal.

Liver function test was repeated after 3 days showing SGPT: 170 U/L, SGPT: 38 U/L, bilirubin (T/D): 0.7/0.55 mg/dl, ALP: 371 U/L, and GGT: 466 U/L. The patient was discharged home to be followed up by liver function test. Two weeks later during regular visit, the patient was found to be jaundiced and started to develop itching with 1 episode of clay-colored stools. So she was readmitted to the hospital. Repeat liver function tests showed a dramatic increase in bilirubin (T/D): 9.13/8.46 mg/dl, ALP: 1373 U/L, and GGT: 1062 U/L. The SGPT and SGOT were 232 and 233 U/L, respectively. The lipase level was 3036 U/L (4–23 U/L), and the amylase level was 226 U/L (3–50 U/L). Repeat ultrasound of the abdomen revealed a normal-sized liver of homogenous structure with dilatation of the intrahepatic and extrahepatic biliary duct with no signs of cholecystitis. The common bile duct (CBD) measured 10 mm with calcified sediment measuring 16 × 8 mm in his distal part, near the head of the pancreas.

**Figure 1:**
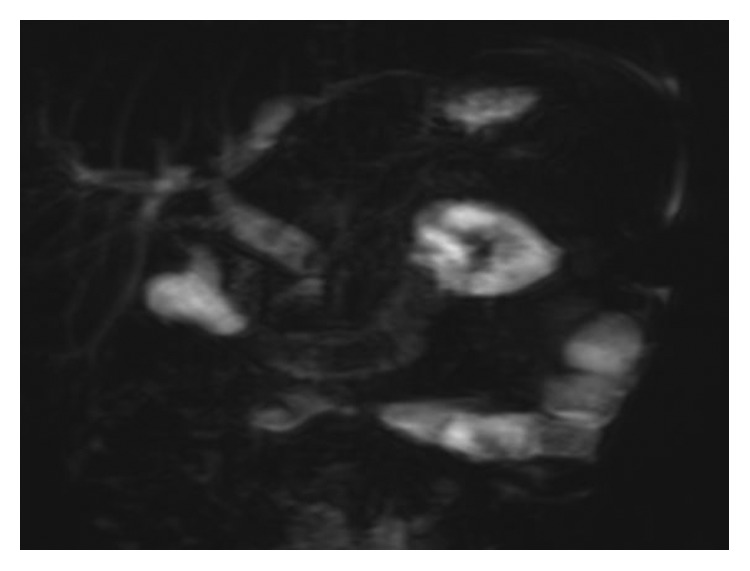
Magnetic resonance cholangiopancreatography (MRCP) showing extrahepatic dilatation of the common bile duct reaching 1.1 cm, with dilatation of the both right and left intrahepatic ducts. The presence of 2 calculi in the distal part of the common bile duct near the head of the pancreas was noticed.

Magnetic resonance cholangiopancreatography (MRCP) showed extrahepatic dilatation with CBD reaching 1.1 cm, with dilatation of the both right and left intrahepatic ducts which confirmed the diagnosis of choledochal cyst (CC) type IVa complicated by biliary stones (Figure 1). The patient was kept NPO and started on IV hydration. Surprisingly, the jaundice improved the day after with normal-colored stools. Repeat LFTs after 48 hours showed marked improvement: SGPT: 129 U/L, bilirubin (T/D): 3.2/2.76 mg/dl, ALP: 951 U/L, GGT: 749 U/L, and lipase: 63 U/L.

A complete resection of extrahepatic ducts with Roux-en-Y hepaticoenterostomy was performed uneventfully. Follow-up after 3 months showed complete normalization of liver enzymes (SGPT: 21 U/L, SGOT: 35 U/L, bilirubin (T/D): 0.25/0.09 mg/dl, ALP: 208 U/L, GGT: 7 U/L, and lipase: 74 U/L).

## 3. Discussion

Epstein–Barr virus infection is common in children, usually presenting as infectious mononucleosis, including fever, tonsillitis, and lymphadenopathy associated with self-resolving increase in transaminases with predominantly cholestatic features. Liver function abnormalities occur more often during the second or third week of the illness, and the liver function returns to normal after 3 to 6 weeks [2]. Transaminases are usually mildly elevated (two or three times the upper normal limit), consistent with parenchymal injury. Although cholestasis is commonly reported in adult population (55 to 65% of the patients), only few cases are reported in children (5% of patients) [1]. The mechanism for the cholestasis in EBV-associated hepatitis remains unknown. A possible explanation is the effect of proinflammatory cytokines which interfere with the activity of both the sinusoidal and canalicular transporting systems that may lead to cholestasis [3].

In our patient, the transaminases were mildly elevated at the beginning of the illness with a cholestatic pattern. However, after 2 weeks from the onset of illness, the patient developed progressive clinical jaundice followed by obstructive jaundice with itching associated with marked increase in GGT and alkaline phosphatase. This was relatively unexpected in the course of EBV infection which is considered so far as benign with progressive decrease in liver function tests after the second week. This is the reason why we repeated the ultrasound of the abdomen to rule out other etiologies such as obstructive stone or choledochal malformation. The ultrasound raised the suspicion of choledochal malformation. Unfortunately, in our patient, the diagnosis was missed on the first ultrasound although the latter is considered as the first modality of choice if performed by an expert ultrasonographer (70–90% of detection) [4]. The diagnosis of choledochal cyst type IVa was confirmed later by MRCP. The choledochal cyst was complicated by biliary stone which explained the recurrent episodes of pancreatitis. To the best to our knowledge and after review of the literature, our case is the first reported case about coinfection of CC with EBV infection. The EBV infection in our patient unmasked the underlying choledochal cyst which was completely asymptomatic before. Knowing that according to the literature, the most common presenting symptom in this age group is abdominal mass with or without clay-colored stools [5]. Our patient was completely asymptomatic before the occurrence of EBV infection.

Management of CC is complete excision of the cyst with Roux-en-Y hepaticoenterostomy. Complete excision of the cyst dramatically decreases the risk of malignant transformation from 26% if left untreated to 0.7–6% after surgery [4]. However, this complex surgery is associated with significant short- and long-term morbidity. Timing of surgery, especially in asymptomatic infants, is difficult. In our patient, we performed a Roux-en-Y hepaticojejunostomy after normalization of the pancreatic enzymes. The short-term follow-up after 6 months from surgery showed complete normalization of liver function tests with resolution of intrahepatic bile duct dilation.

In conclusion, the development of clinical jaundice in EBV infection in the pediatric age group needs close follow-up. In case of nonamelioration or exacerbation of the symptoms, another diagnosis should be addressed. This highlights the importance of close monitoring of liver function tests in these patients in order not to miss a serious underlying condition such as choledochal malformation.
